# A Concomitant Intramyocardial and Pulmonary Hydatid Cyst: A Rare Case Report

**DOI:** 10.21470/1678-9741-2016-0046

**Published:** 2017

**Authors:** Harsh Sateesh Seth, Prashant Mishra, Jayant V. Khandekar, Chaitanya Raut, Chandan Kumar Ray Mohapatra, Ganesh Kumar K. Ammannaya

**Affiliations:** 1 Lokmanya Tilak Municipal Medical College & General Hospital, Sion West, Mumbai, Maharashtra, India.

**Keywords:** Echinococcosis, Pulmonary, Heart Diseases/surgery

## Abstract

Cardiac hydatid cyst is an uncommon but potentially fatal disease. In cystic *Echinococcus* humans are an accidental host. Liver and lungs are the most frequently involved organs. Herein a unique case of intramyocardial hydatid cyst of left ventricle along with pulmonary hydatid cyst in a 38-year-old lady is reported. Surgical removal of the cardiac hydatid cyst was done with the aid of cardiopulmonary bypass followed by removal of pulmonary hydatid cyst.

**Table:** 

Abbreviations, acronyms & symbols
ECG = Electrocardiogram

## INTRODUCTION

Hydatid cyst is a human parasitic disease caused by larval stage of *Echinococcus granulosus.* It is endemic in countries where farm animals are raised^[1]^. Cardiac hydatidosis has been reported infrequently even in countries where hydatid disease is endemic: 0.5-2% in comparison with liver (65%) and lungs (25%)^[2,3]^. The diagnosis is difficult because of long latency between infection and manifestation of disease. Hence we report a case of a young lady with intramyocardial and pulmonary hydatid cyst, which was surgically removed.

## CASE REPORT

A 38-year-old lady was admitted to our hospital with hemoptysis followed by chest pain, breathlessness and lowgrade fever. There was history of rearing pet animals. On clinical evaluation, no abnormal findings were detected. Her hematological and biochemical parameters were within normal limits except ELISA test for *Echinococcus granulosus,* which was positive.

**Fig. 1:**
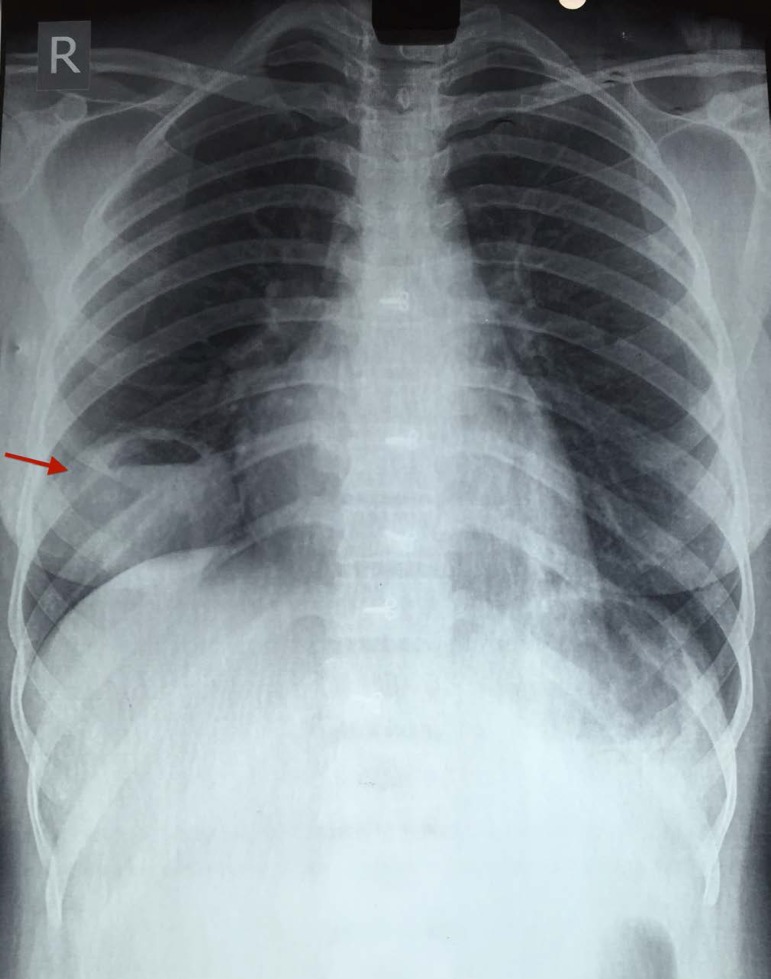
PA chest radiography view showing a well-defined lesion (arrow) in the right lower zone of the lung with air fluid level.

Electrocardiogram (ECG) showed T-wave inversion in V2, V3, V4, and V6. The chest radiography showed well defined lesion in the right lower zone of the lung with air fluid level, and normal cardiac silhouette (Figure 1). Ultrasonography of the abdomen was normal. Two-dimensional echocardiography showing a lesion measuring 2 × 1.8 cm cystic mass was noted at the apex (intramyocardial). Chest high resolution computed tomography showed a cystic lesion with air fluid level measuring 5.7×4.5×5 cm in the subpleural region of the right lower lobe of the lung. Cardiac magnetic resonance imaging confirmed an intramyocardial hydatid cyst at the apex of the left ventricle.

**Fig. 2:**
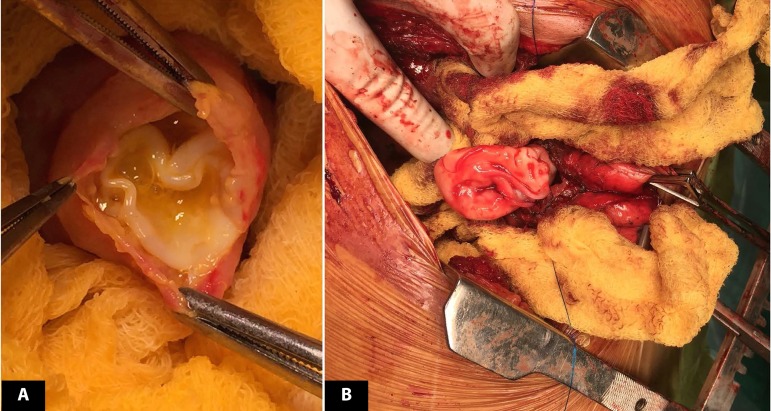
Intraoperative photograph of intramyocardial left ventricular hydatid cyst (A) and pulmonary hydatid cyst (B).

Median sternotomy was done for removal of both cardiac and lung hydatid cyst. Cystectomy was performed under aortobicaval cardiopulmonary bypass with arrested heart. Cystic lesion of size 2x 2 cm was found to be encapsulated by a well-formed myocardium at the apex of left ventricle. The entire pericardial cavity was covered with mops soaked in hypertonic saline. The cystic cavity was sterilized with 5% cetrimide and fluid was aspirated. The cyst cavity was then opened and the daughter cyst was removed in Toto (Figure 2). The edge of the cyst was excised and the cavity was closed with 5-0 polypropylene plegeted continuous sutures over Teflon felt. The pulmonary hydatid cyst was removed via right posterolateral thoracotomy with adequate precaution using scolicidal agents around and by giving positive pressure ventilation (Figure 2). Histopathological study was consistent with hydatid cyst. The patient had an uneventful postoperative course and was discharged on Tab Albendazole 400 mg twice a day for six months. The patient was asymptomatic in the eight-month follow-up period after surgery.

## DISCUSSION

Hydatidosis is a parasitic infection caused by *Echinococcus granulosus.* The life cycle of this tapeworm involves dogs and other canids as definitive hosts, and domestic and wild ungulates, usually sheep, as intermediate hosts. Human beings are only incidental intermediate hosts. The infection, often acquired in childhood during play with infected dogs, is most common in sheep-raising areas of the world^[1]^. Hydatid cyst of the heart is an uncommon lesion. The most frequent locations of hydatid cysts in humans are the liver 65% and lungs 25%, with only 0.5-2% of cases located in the heart^[2,3]^. The common location of cardiac hydatid cysts are the left ventricle (55-60%), right ventricle (15%), interventricular septum (9%), left atrium (8%), right atrium (4%) and interatrial septum (2%)^[2-4]^.

After infection, the embryo usually reaches the myocardium via the coronary circulation from the left side of the heart. The cyst is then formed within a period of one to five years^[4]^. Clinical presentation of cardiac hydatid cyst is variable; the usual manifestations reported are dyspnea, chest pain and palpitations^[4]^. The most dangerous and potentially lethal complication of cysts is their intracardiac or intrapericardial rupture, which may result in anaphylactic shock, embolization, or cardiac tamponade, acute pericarditis, and chronic constrictive pericarditis^[4]^. Cysts growing toward the epicardium can compress the small coronaries, presenting with features of coronary artery disease. Involvement of interventricular septum may lead to conduction defects.

Pulmonary hydatid disease affects the right lung in around 60% of cases, 30% exhibit multiple pulmonary cysts, 20% bilateral cysts and 60% are located in lower lobes^[5]^. Pulmonary cysts typically increase in diameter at 1-5 cm/yr. Most lung cysts are incidentally diagnosed on chest radiographs. Occasionally, patients present with cough, hemoptysis, or chest pain^[5]^. Complication of pulmonary hydatid cysts are mainly due to the release of antigenic material and secondary immunological reactions that develop cyst rupture.

The diagnosis is difficult because of long latency between infection and manifestation of disease. The chest radiograph may reveal cardiomegaly, and meniscus sign for pulmonary hydatid^[6]^. ECG may show T-wave inversion, premature ventricular beats. Hematological profile may reveal eosinophilia of varying degree. Serological tests are positive in only about 50% of patients and thus limited diagnostic accuracy^[2]^. Echocardiography remains the most reliable diagnostic measure for cardiac hydatid cyst^[4]^. Computed tomography and magnetic resonance imaging are other valuable diagnostic tools.

The recommended treatment is enucleation, pericystectomy or cystectomy with cappitonage of the cyst under cardiopulmonary bypass via median sternotomy with topical scolicidal agents in the surrounding operative field^[2-4]^. The operative management for concomitant pulmonary hydatid cyst can be done in the same setting or different setting. In our case, it was done in the same setting via separate right posterolateral thoracotomy.

During surgery, it is important to minimize spillage of the cystic contents in order to prevent intraoperative dissemination and eventual recurrence. This may be accomplished by delivery of intact cyst or by cystic fluid aspiration with use of scolicidal agent and preoperative therapy with Albendazole. As a rule, the heart should not be manipulated before application of cross clamp and cardiopulmonary bypass. Prior to enucleation, a hydatid cyst is usually sterilized by instillation of scolicidal agents such as 2% formalin, 0.5% silver nitrate solution, 20% hypertonic saline solution, 1% iodine solution or 5% cetrimide solution. We used 5% cetrimide for instillation and soaked mops around the cyst cavity^[2-4]^. Though cardiac hydatid cysts be very uncommon, the possibility of this disease should be kept in mind especially in endemic areas. In order to prevent fatal cardiac complications, cardiac hydatid cyst should be removed even in asymptomatic patients.

**Table:** 

Authors' roles & responsibilities	
HSS	Conception and study design; manuscript redaction or critical review of its content; final manuscript approval
PM	Conception and study design; manuscript redaction or critical review of its content; final manuscript approval
JVK	Conception and study design; final manuscript approval
CR	Critical review of its content; final manuscript approval
CKRM	Final manuscript approval
GKKA	Final manuscript approval
